# Paralysis—Cerebral, Bulbar and Spinal

**Published:** 1886-12

**Authors:** 


PARALYSES—CEREBRAL, BULBAR AND SPINAL. A manual of diagnosis for students and practitioners. By H. Charlton Bastian, M. A., M. D., F. R. S., Fellow of the Royal College of Physicians; Examiner in Medicine at the Royal College of Physicians; Professor of Clinical Medicine and of Pathological Anatomy, University College, London; Physician to University College Hospital, and to the National Hospital for the Paralyzed and Epileptic; Crown Referee in cases of supposed insanity. With numerous illustrations. New York: D. Appleton & Co., 1, 3 and 5 Bond street. 1886.

The multiplicity of paralytic affections of different kinds from various causes which are found among all classes of people in this country renders the presentation of this subject most opportune at present.

The discussion of recent developments touching the localization of motor centres receives much additional interest by the report of four successful results in operating for the removal of tumors or other abnormal modifications of the brain by Mr. Victor Horsly within a recent period. These important steps in cerebral surgery were based upon observation of the peripheral disturbances connected with the motor centres of the brain and indicate a progressive stride in practical neurology, while they redound to the success of operative measures, and the great distinction of the noted surgeon who has thus conferred this crowning glory upon modern surgery does honor to Great Britain.

A satisfactory demonstration of the anatomy and physiology of the brain and spinal cord, with a clear outline of the functions of the nerves, affords a good basis for understanding the full details which are given in illustration of nervous disorders. The figures assist greatly in comprehending the descriptions of the lesions and modifications of the brain that pertain to the minute structure of its various divisions noted in this work.

It is properly observed by the author in his preface, that the various forms of paralysis from brain disease, from disease of the bulb, and from disease of the spinal cord, are now so numerous, and so many recent advances have been made to our knowledge in this direction, that some such aid to diagnosis may well be looked for by those for whom this work is intended.

The book consists of four main general divisions under the headings of encephalic paralysis, paralysis of bulbar origin, paralysis due to lesions of the cranial nerves, and paralysis of spinal origin, with a closing chapter on combined regional and pathological diagnosis, which are worthy of the reader's careful study.

